# Ovariotomy

**Published:** 1862-12

**Authors:** 


Ovariotomy may be truly said to be the operation on the order of the day. Mr. S. Wells, on showing some ovarian tumors at the Pathological Society, remarked that "although last session he reported 12 cases with 7 deaths, he could now say that, reckoning the above recent cases, the last 9 were successful operations." On the 15th Oct., Mr. Bryant removed a large ovarian cyst from a woman in Guy's Hospital. The patient has, we understand, gone on well to a recovery, no bad symptoms having appeared. Another operation of this nature has been performed at St. Bartholomew's, and one some few weeks ago at St. George's Hospital. In France, also, in all quarters, ovariotomists are springing up.—*Brit. Med. Jour.*

